# Cognitive training incorporating temporal information processing improves linguistic and non-linguistic functions in people with aphasia

**DOI:** 10.1038/s41598-023-41045-0

**Published:** 2023-08-28

**Authors:** Mateusz Choinski, Magdalena Stanczyk, Aneta Szymaszek

**Affiliations:** 1 Laboratory of Neurophysiology of Mind, BRAINCITY-Center of Excellence for Neural Plasticity and Brain Disorders, Nencki Institute of Experimental Biology, Polish Academy of Sciences, Warsaw, Poland; 2 Faculty of Psychology, University of Warsaw, Warsaw, Poland

**Keywords:** Human behaviour, Language

## Abstract

People with aphasia (PWA) often present deficits in non-linguistic cognitive functions, such as executive functions, working memory, and temporal information processing (TIP), which intensify the associated speech difficulties and hinder the rehabilitation process. Therefore, training targeting non-linguistic cognitive function deficiencies may be useful in the treatment of aphasia. The present study compared the effects of the novel Dr. Neuronowski^®^ training method (experimental training), which particularly emphasizes TIP, with the linguistic training commonly applied in clinical practice (control training). Thirty four PWA underwent linguistic and non-linguistic assessments before and after the training as well as a follow-up assessment. Patients were randomly assigned to either experimental (n = 18) or control groups (n = 16). The experimental training improved both non-linguistic functions (TIP and verbal short-term and working memory) and linguistic functions: phoneme discrimination, sentence comprehension, grammar comprehension, verbal fluency, and naming. In contrast, the control training improved only grammar comprehension and naming. The follow-up assessment confirmed the stability of the effects of both trainings over time. Thus, in PWA, Dr. Neuronowski^®^ training appears to have broader benefits for linguistic and non-linguistic functions than does linguistic training. This provides evidence that Dr. Neuronowski^®^ may be considered a novel tool with potential clinical applications.

## Introduction

Stroke is the second leading cause of death and disability in the world^1^. One of the most common consequences of stroke is aphasia, which affects at least one third of stroke survivors^2^ Aphasia is a communication disability due to acquired impairment of language modalities resulting from a brain lesion in the language-dominant hemisphere^3,4^. Depending on the region of the brain in which the lesion occurs, people with aphasia (PWA) present difficulties with speech production and/or comprehension accompanied by deficient writing and reading. In addition to linguistic impairments (the most salient symptoms of aphasia), PWA often exhibit difficulties with non-linguistic cognitive functions, such as attention^5,6^, executive functions^7,8^, and memory^9,10^. These cognitive deficits are often reported to occur independently of the verbal stimuli used to assess them (e.g.,^11–14^). However, as communication skills are dependent on the abovementioned functions, such impairments may potentially intensify language deficits in PWA and impede the process of aphasia rehabilitation and language function restoration^15^.

PWA also have difficulties with temporal information processing (TIP). Some studies have demonstrated a link between TIP and language. Human speech is constrained by temporal organization in the millisecond and multisecond time domains. Millisecond TIP is related to phonological encoding/decoding and syllabification, while multisecond TIP is involved in lexical selection, sentence production, and perception^16,17^. In the multisecond domain, the spontaneous flow of speech is temporally segmented and chunked into phrases limited in time up to a few seconds separable by pauses. This segmentation plays a crucial role by allowing the speaker to prepare the next phrase and the listener to process the incoming information^18^. Several authors have indicated that PWA demonstrate deficits on different TIP levels depending on their aphasic symptoms^17^. Thus, patients with left hemispheric lesions and Broca’s aphasia, who present remarkable difficulties with speech production, have TIP deficits on the multisecond level compared to patients with right-hemisphere lesions, healthy controls, or people with Wernicke’s aphasia^19,20^. Authors conclude that impairment in fluent speech and sentence construction may derive from deficient temporal integration on the level of a few seconds^16^.

The present study focused mainly on millisecond TIP which seems essential for speech perception, especially for the processing of basic units of language—phonemes (e.g., stop-consonants limited in time up to ca. 40 ms^21^). Specifically, the differentiation of voiced and unvoiced stop-consonants in syllables (like Do and To) is dependent on the Voice-Onset-Time, i.e., the time between the burst of air and the start of laryngeal pulsing. The Voice-Onset-Time phenomenon is further evidence for the millisecond range being fundamental for speech perception^16,22^.Thus, efficient TIP in the millisecond range may be crucial for correctly encoding and decoding phonological forms.

The efficiency of millisecond TIP is determined by patients’ ability to accurately identify and process the order of stimuli that occur in rapid succession. It is often measured with the auditory Temporal-Order Judgment (TOJ) paradigm^23–25^. This paradigm can be used to assess temporal-order threshold (TOT), which is defined as the shortest time gap between two sounds (in the auditory TOJ paradigm) at which the participant is able to report the before–after relation of those sounds. Lower threshold values reflect more efficient millisecond TIP. Several studies have indicated that, for normal healthy volunteers, this gap usually ranges from ca. 30 ms up to 80 ms^26–29^. Elevated thresholds in the detection of temporal order compared to control participants have been identified in people with Wernicke-like deficits (i.e., in phonemic hearing and auditory comprehension). Wittmann et al.^20^ reported that participants with left-hemispheric lesions that overlap in the supramarginal gyrus, angular gyrus, middle temporal gyrus, and superior temporal gyrus are characterised by elevated auditory TOTs. These regions are known to participate in phoneme processing and speech comprehension^30–33^.

According to previous studies, TIP may be also considered as a neural frame for many non-linguistic cognitive functions that are characterised by specific temporal dynamics in the millisecond range^34^. This is in line with the taxonomy of cognitive functions proposed by von Steinbüchel and Pöppel^35^, who suggested that cognitive functions may be divided into content-related functions (“what” functions) and logistic functions (“how” functions). Content-related functions include perception, memory, and language. On the other hand, TIP is considered a logistic function, which constitutes the neuronal template of our abovementioned content-related functions. Numerous studies have shown that TIP is positively associated with planning^36^, short-term and working memory^37,38^ as well as motor behaviours^39^, which provides evidence for von Steinbüchel and Pöppel’s model.

Many speech and language trainings that directly train impaired linguistic functions are recognized as standard elements of rehabilitation^40^. However, as mentioned above, linguistic functions in PWA are frequently associated with parallel impaired non-linguistic ones. In this context, some researchers have investigated whether cognitive trainings improve language competency in PWA^41–44^. For example, Zakarias et al.^41,42^ applied training based on the n-back task using verbal and non-verbal stimuli and reported improved sentence comprehension. Nikravesh et al.^44^ noticed significant improvements of PWA in language performance, i.e., speech fluency, naming, repetition and auditory comprehension following working memory training. Furthermore, Salis^45^ reported the effectiveness of a short-term memory treatment using a serial word recognition task in improving sentence comprehension in a patient with severe aphasia. This effect was, however, not replicated in the further study of Salis et al.^43^ involving five PWA. The generalisation of memory training outcomes to various aspects of language processing could be interpreted as indicating far-transfer effects—that is to say, the transfer of training benefits to a different domain that shares underlying mental processes with the trained domain^46,47^.

Some pilot studies investigating the effects of millisecond TIP trainings in PWA have been conducted. Szymaszek et al.^48^ compared the effects of an experimental training based solely on the perception of the temporal order of two sounds presented in rapid succession with a nontemporal control training based on differentiating the loudness of two sounds. After eight sessions of the experimental training, improved TIP was observed. Moreover, a transfer of improvement was noticed in untrained linguistic functions (sentence comprehension and voice/unvoiced contrast detection), while the control training did not result in any changes in either TIP or in linguistic functions. Similar results were obtained in another study^21^, which provided evidence that several functions improved following TIP training: the directly-trained millisecond TIP, language functions such as sentence comprehension and phoneme discrimination, as well as some cognitive functions (working memory and attention). The control group improved only in phoneme discrimination. The abovementioned studies on the effectiveness of TIP training in aphasia demonstrate well-replicated far-transfer effects on untrained linguistic functions. This complements the previously-described role of TIP in language functioning, as well as the coexistence of TIP and linguistic deficits in PWA.

Dr. Neuronowski^®^ is a novel multimodal computer software treatment method for PWA that focuses on a variety of cognitive functions, with a particular emphasis on TIP. The current study investigated the effectiveness of the Dr. Neuronowski^®^ treatment method, comparing it with a computer-based linguistic therapy software as a control. This enabled us to directly compare the effects of Dr. Neuronowski^®^ training with exercises more traditionally used in aphasia rehabilitation. Moreover, the benefits of training were assessed with extended diagnostic procedures that focused on TIP and language as well as other cognitive functions that are also temporally segmented in the millisecond domain and are strongly related to language skill.

## Methods

### Participants

Thirty four post-stroke patients (22 male) suffering from aphasia after their first left-hemispheric stroke (lesion age: Me = 32 weeks; min–max: 5–195 weeks) participated in the study. Participants’ ages ranged from 30 to 82 years (M ± SD: 59 ± 13 years). They were right-handed native speakers of Polish. Participants displayed normal hearing verified by pure-tone screening audiometry (Audiometer MA33, MAICO). The following exclusion criteria, verified during an interview with the caregivers of the patients, were applied: recurrent stroke, global aphasia, severe comprehension impairment, post-stroke visual deficits, prior neurological or psychiatric diseases, substance abuse, history of head injuries, and signs of dementia. Detailed characteristics for the patients are presented in Table 1.

**Table 1:** Characteristics of the patient sample (*M* male, *F* female, *n.a.* not available, *I* ischemic stroke, *H* haemorrhagic stroke).

Number	Age (years)	Sex	Lesion age (weeks)	Lesion volume (mm^3^)	Type of stroke	Group	Follow-up assessment
1	82	F	13	69,227	I	Experimental group (ExpG)	Yes
2	75	F	47	n.a	I		Yes
3	62	F	31	56,184	I		No
4	48	F	20	n.a	I		No
5	44	F	25	98,472	I		No
6	72	F	114	111,584	I		No
7	67	M	19	165,650	I		Yes
8	60	M	67	131,755	I		Yes
9	60	M	8	n.a	I		Yes
10	48	M	9	45,655	I		Yes
11	69	M	21	56,011	I		Yes
12	64	M	10	84,121	I		Yes
13	58	M	169	53,182	I		No
14	78	M	5	n.a	I		No
15	43	M	49	121,210	I		No
16	51	F	37	44,781	H		Yes
17	42	M	28	n.a	H		Yes
18	62	M	33	76,228	H		No
19	58	F	72	93,033	I	Control group (ConG)	Yes
20	54	F	194	103,136	I		Yes
21	49	F	96	71,275	I		No
22	50	F	195	168,693	I		No
23	68	M	73	n.a	I		Yes
24	67	M	57	157,949	I		Yes
25	49	M	191	n.a	I		Yes
26	60	M	81	73,481	I		Yes
27	46	M	18	17,216	I		Yes
28	78	M	8	55,118	I		No
29	40	M	73	95,664	I		No
30	51	M	12	42,429	I		No
31	74	M	6	37,303	I		No
32	71	M	20	140,928	I		No
33	62	F	67	69,760	H		Yes
34	30	M	19	33,579	H		Yes

This study was controlled, randomised, and single-blinded. The patients were classified into two groups: the experimental group (ExpG), who used the experimental training program, and the control group (ConG), who used the control training. For a detailed description of both trainings, see below. As PWA usually display huge inter-individual variability, ExpG and ConG were matched as closely as possible for age, lesion age and volume, gender, as well as pre-training levels of tested functions (i.e., speech difficulties, TIP, memory, and executive functions). It is worth noting that even though some of the abovementioned cognitive variables tended to be higher and lesion age tended to be shorter in ExpG, the statistical differences were nonsignificant. Descriptions of particular assessment procedures can be found in the Procedure section and a statistical comparison of the groups before training can be found in Table 2.

**Table 2:** Characteristics of the two training groups in the pre-training assessment: mean and standard deviation values of demographic variables and scores of tests.

	ExpG (n = 18) M (SD)	ConG (n = 16) M (SD)	Comparison
**Demographic variables**			
Age (years)	60.28 (12.30)	56.69 (13.02)	t(32) = -0.827 p = .415
Lesion age (weeks)	39.17 (41.67)	73.88 (66.09)	U = 100.0 p = .129
Lesion volume (mm^3^)	85,697 (37,704)	82,826 (46,959)	t(25) = -.174 p = .863
Gender (male/female)	11/7	11/5	χ^2^(1) = 0.216 p = 0.642
**Pre-training assessment results**			
Speech comprehension			
Sentence comprehension (/50)	33.69 (12.19)	26.58 (13.17)	U = 55.5 p = 0.220
Grammar comprehension (/16)	10.56 (3.7)	9.56 (4.29)	t(32) = − 0.725 p = 0.474
Word comprehension (/14)	11.44 (2.83)	11.94 (2.46)	U = 131.0 p = 0.645
Phoneme discrimination (/25)	20.78 (4.05)	19.38 (4.47)	U = 116.5 p = 0.340
Speech production			
Naming (/13)	5.56 (4.21)	3.79 (3.77)	U = 87.5 p = 0.303
Verbal fluency (number of words)	10.56 (7.29)	9.31 (7.09)	U = 88.0 p = 0.482
TIP			
TOJ (ms)	166.24 (87.23)	122.58 (50.39)	U = 91.0 p = 0.105
Memory			
Verbal short-term memory (score)	3 (2.56)	4.2 (2.88)	U = 89.0 p = 0.215
Spatial short-term memory (score)	6.0 (2.32)	6.75 (2.54)	t(31) = 0.89 p = 0.382
Executive functions			
Planning ability (/14)	3.47 (1.92)	3.93 (2.15)	U = 99.5 p = 0.584
Verbal working memory (score)	2.75 (2.49)	2.73 (2.37)	U = 115.5 p = 0.856
Spatial working memory (score)	5.47 (2.90)	5.31 (2.75)	t(31) = − 0.161 p = 0.873

**Figure 1:**
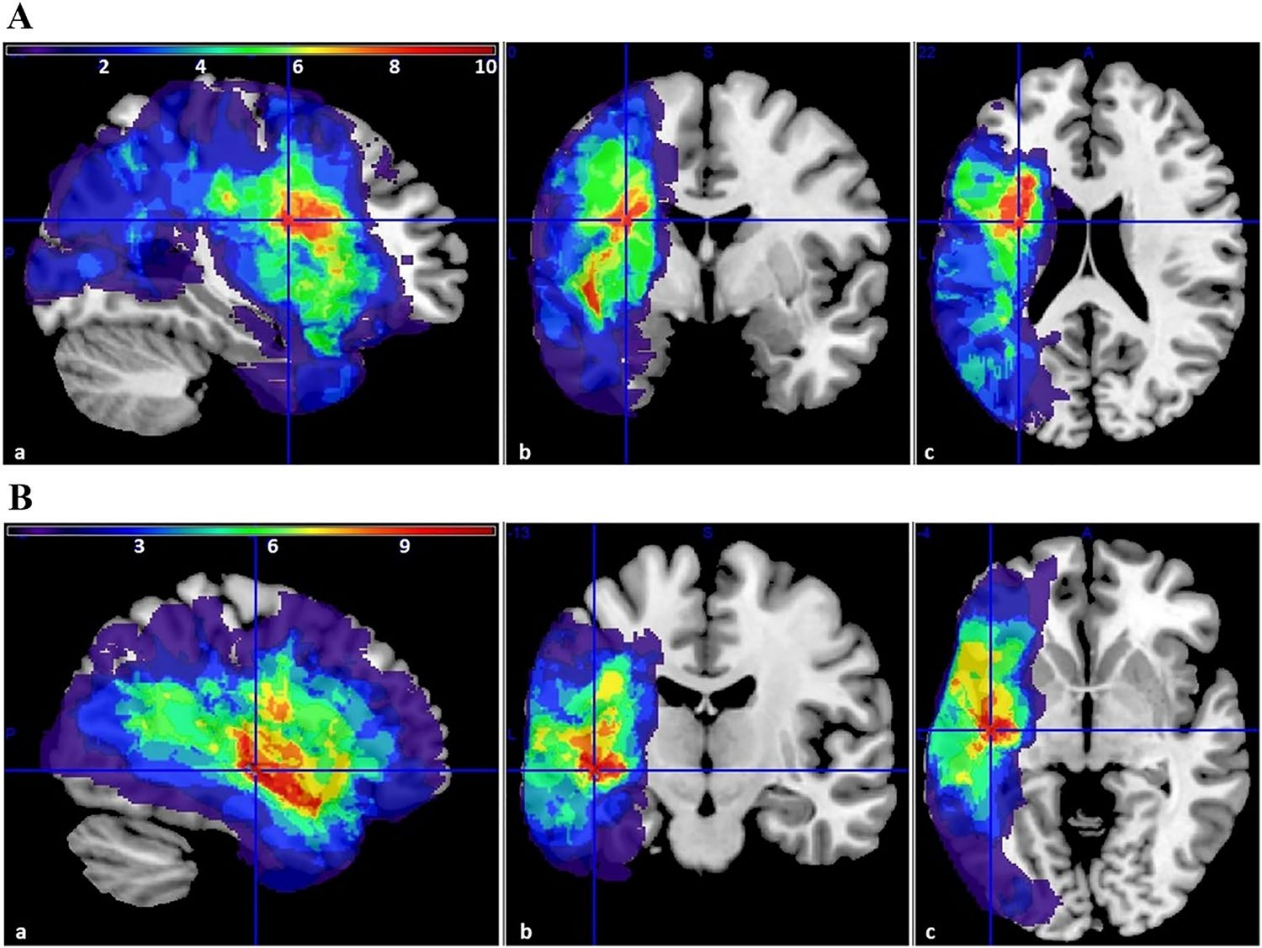
Lesion overlap maps of participants from (**A**) ExpG and (**B**) ConG. Brighter colours (yellow, orange, and green) indicate a greater number of participants with lesions in particular regions. Darker colours (blue and green) indicate regions of less overlap. The overlays are presented in three sections: (**a**) sagittal, (**b**) coronal, and (**c**) axial.

The location of the lesion was verified by CT or MRI in 27 out of 34 individuals (13 from ExpG, 14 from ConG; Fig. 1). Neuroanatomical analyses using MRIcroN and SPM12 confirmed that lesions in both groups were localised only in the left hemisphere. In ExpG, the lesion mainly affected the insula, central operculum, precentral gyrus, and planum temporale. In ConG, the lesion mainly affected the central operculum, planum polare, insula, postcentral gyrus, Heschl’s gyrus, and planum temporale.

### Procedure

**Figure 2:**
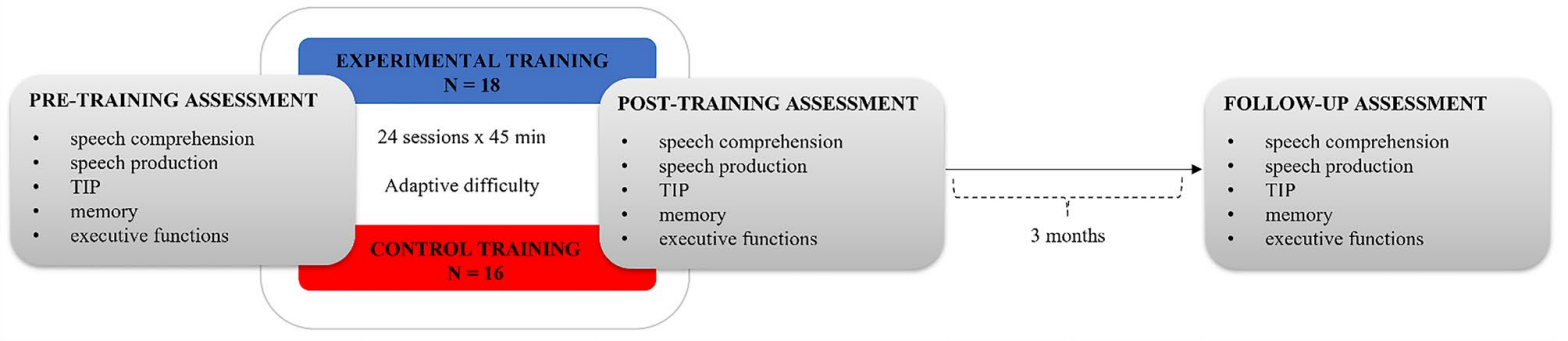
Schema of the study.

The study was comprised of both assessment and training procedures. The assessment procedures consisted of several neuropsychological measurements evaluating speech comprehension and production, TIP, memory, and executive functions (Fig. 2; see below for description of particular procedures). The assessment procedures were conducted three times: before training (pre-training assessment), after training (post-training assessment), and at a follow-up ca. 3 months after the training. Only 19 patients (10 from the experimental group and 9 from the control group; Table 1) took part in the follow-up assessment. During the 3 months between the training and follow-up, the remaining 15 patients either suffered from additional medical incidents that may affect long-term language recovery (i.e., recurrent stroke, epileptic seizures), and therefore could not be considered in the follow-up assessment, or it was not possible to perform the scheduled follow-up at 3 months due to COVID lockdowns. The schema of the study design is displayed in Fig. 2.

### Assessment procedures

*Speech comprehension* was assessed with sentence comprehension, grammar comprehension, word comprehension, and phoneme discrimination.

- Sentence comprehension was assessed with a Token Test (Aachener Aphasia Test battery^49^), consisting of 50 spoken commands classified into five sections of increasing length and complexity. The task was to follow the commands (e.g., *“Touch the white circle after taking away the yellow square”*) either by pointing to or manipulating plastic tokens (coloured squares and circles of two sizes: big / small).
  Outcome measure: number of correct responses.
- Grammar comprehension was assessed with the Grammar-Sentence Comprehension Test^50^. Participants listened to 16 sentences. During each sentence (e.g., “*The duck is flying above the tree”*) the participant was presented with a set of four pictures on a response card indicating four different syntactic situations (e.g., above, next to, behind, or under). The task was to indicate the picture corresponding to the situation in the sentence heard.
  Outcome measure: number of correct responses.
- Word comprehension was assessed with the Vocabulary-Word Comprehension Test^50^, consisting of cards with four different pictures each from the same semantic category. The task was to point to the picture of a given object or action. Of 14 test trials, seven consisted of action verbs (e.g., to crawl) and the next seven of object names (e.g., cauliflower).
  Outcome measure: number of correct responses.
- Phoneme discrimination was assessed with the Phoneme Hearing Test (Battery of Phonological tests^51^). This test consists of 25 paired pseudowords (18 pairs differing in terms of the place/manner of articulation or voicing; seven pairs were the same). The task was to indicate by pointing to response cards (Yes/No) whether the heard pair included the same or different pseudowords.
  Outcome measure: number of correct responses.

*Speech production* was assessed with naming and verbal fluency.

- Naming was assessed with the Vocabulary-Word Production Test^50^, which consists of 13 pictures of objects (e.g., koala) or actions (e.g., jump). The task was to name the object or action using a single word.
  Outcome measure: the number of correct responses.
- Verbal fluency was measured with the Semantic Verbal Fluency Test in two categories (animals or fruits)^52^. The task was to produce, in one minute, as many words as possible from each category.
  *Outcome* measure: number of correctly produced nouns.

*Temporal information processing* was assessed with a Temporal Order Judgement (TOJ) task^23^. Participants were presented with pairs of two rectangular pulses (clicks) of 1 ms duration each with varied inter-stimulus intervals. The clicks were presented monaurally—one click was presented to one ear, followed by a second click to the other ear. The stimuli were delivered through Sennheiser HD 201 headphones. The participant’s task was to report the order of the paired clicks by pointing to their ears in the order that the clicks appeared. Two alternative responses were possible: left–right or right–left. The intervals between clicks in a pair varied from 1 to 600 ms, according to an adaptive maximum-likelihood-based algorithm^53^. Each interval was calculated on the basis of correctness achieved in previous trials and adjusted with ‘Yet Another Adaptive Procedure^54^ on the basis of maximum likelihood parameter estimation. Individual TOT was estimated as the minimum interval at which a participant reported the order of the clicks with 75% correctness. Measurement continued until the TOT value was located with a probability of 95% inside a ± 5 ms interval around the currently estimated threshold.

Outcome measure: TOT value in milliseconds. 

*Memory* was assessed in verbal short-term memory and spatial short-term memory.

- Verbal short-term memory was assessed with the Verbal Memory Test Forwards (Verbal Memory Test^55^), which consists of nine concrete unrelated monosyllabic words (in Polish: “*kot, smok, sok, płot, młot, koc, nos, noc, blok”,* in English: “*cat, dragon, juice, fence, hammer, blanket, nose, night, building”*) and a set of nine pictures corresponding to these words. After verifying that participants were able to correctly match the words with the appropriate pictures, the task was to reproduce (by pointing to the appropriate pictures) the sequence of words in the same order as read by the experimenter. The test difficulty in subsequent steps was increased from two words in the first sequence up to a maximum of nine words in the last sequence.
  Outcome measure: score—the number of correctly reproduced sequences of words.
- Spatial short-term memory was assessed with the Corsi Block Tapping Test Forwards (Vienna Test System^56^). The participant was presented with a matrix of nine identical blocks. The task was to tap the blocks in the same order as previously indicated by the cursor. The test difficulty in subsequent steps was increased from three blocks in the first sequence up to the maximum eight blocks in the last sequence.
  Outcome measure: score—the number of correctly reproduced sequences.

*Executive functions* were assessed in terms of planning ability and verbal working memory and spatial working memory.

- Planning ability was assessed with the short form of the Tower of London—Freiburg Version task (Vienna Test System^56^). The task consisted of two boards, each containing three balls of different colours placed on three rods of different height. The task was to replicate on the lower board the configuration of ball placement presented on the upper board in the minimum number of moves. The test difficulty increased in consecutive trials with an increasing minimum number of moves, varying from three to six.
  Outcome measure: total number of problems solved in the minimum number of moves.
- Verbal working memory was assessed with a Verbal Memory Test Backwards^55^. This test is the second part of the Verbal Memory Test described above. The task was to reproduce the words in the order opposite to that presented by the experimenter by pointing to the appropriate picture. The test difficulty increased from two words in the first sequence up to a maximum of eight words in the last sequence.
  Outcome measure: score—the number of correctly reproduced sequences of words.
- Spatial working memory was assessed with Corsi Block-Tapping Test Backwards (Vienna Test System^56^). This test is the second part of the Spatial Memory Test described above. The task was to tap the blocks in the order opposite to that indicated by the cursor. The test difficulty increased from three blocks in the first sequence up to a maximum of eight blocks in the last sequence.
  The outcome measure: score—the number of correctly reproduced sequences.

### Training procedure

The *experimental training* used Dr. Neuronowski^®^^57^—a novel multimedia therapeutic software designed at the Nencki Institute and tailored to PWA. The software consists of 31 therapeutic games that simultaneously train several cognitive domains, with a strong emphasis on TIP. The therapeutic games are divided into nine modules. The majority of games involve TIP in the millisecond range, sequencing abilities, and duration judgement. Moreover, Dr. Neuronowski^®^ is extended by exercises training other cognitive functions, i.e., working memory, attention, executive functions, and voice onset time (for detailed description of modules see Table 3).

**Table 3:** Overview of Dr. Neuronowski^®^ modules.

Module	Trained functions
Module 0	Introductory games to familiarise patients with the training, types of games, and sounds used Auditory perception Reaction speed Response inhibition Alertness
Module 1	Auditory perception of nonverbal stimuli Short-term nonverbal auditory memory Selective attention Sustained attention
Module 2	Millisecond TIP—perception of the order of two sounds presented either monaurally or binaurally with various inter-stimulus intervals
Module 3	Millisecond TIP—processing of short sounds presenting in rapid succession Nonverbal and verbal auditory short-term memory—memorising sequences of various length
Module 4	Executive functions—planning, switching, inhibitory control
Module 5	Millisecond TIP—processing of rapidly changing sounds Phonemic hearing based on phoneme change detection in syllables or in words differing in single consonant sounds Verbal auditory short-term memory based on artificially slowed verbal stimuli
Module 6	Verbal auditory short-term memory based on listening to stories with an artificially slow speech rate
Module 7	Millisecond and multisecond TIP—judgement of the duration of short sounds
Module 8	Millisecond and multisecond TIP—estimation of time intervals Delay of responses Response inhibition
Module 9	Phonemic hearing based on artificially modified voice onset time Millisecond TIP—voice onset time perception

The task difficulty in particular games was modified by: number, length, and presentation rate of verbal and nonverbal stimuli, the rate of modified speech, various inter-stimulus intervals in sequentially presented stimuli, application of distractors, and time limits for the patient’s responses.

The *control training* focused on exercises for improving impaired language functions. It was based on multimedia speech-therapy software available on the Polish market: AFASystem^58^ Logopedic Games (Logopedyczne Zabawy^59^), and Phonation—Training of Correct Speech (Dźwięczność—trening poprawnej wymowy^60^). The games involved picture naming (nouns, verbs, adjectives) and speech comprehension (of words, sentences, and longer sections). The task difficulty in following games was determined by word length and frequency, length of linguistic units (words vs sentences vs longer sections), and the presence or absence of cues (initial letter of the word). In contrast to the experimental training, these tasks did not involve any TIP.

The protocols of both the experimental and control trainings involved 24 individual sessions of 45 min each, 3 times a week. All exercises were performed on a tablet. The training sessions were conducted with the assistance of a therapist, whose role was to monitor the patient’s performance and to provide technical assistance in handling the tablet. In both experimental and control trainings, the task difficulty in particular games changed adaptively on the basis of the actual level of the patient’s performance.

### Statistical analyses

To verify the distributions of the resultant data, the Shapiro–Wilk Test was used and further statistical analyses were adjusted accordingly.

To verify the effects of each training type on particular cognitive functions (*post-* vs *pre-training* performance), either the Wilcoxon Signed-Rank Test for two dependent samples (if any variables deviated significantly from the Gaussian distribution) or a mixed-design ANOVA (if all variables were normally distributed) was performed.

To examine the stability of training effects *(follow-up* vs *post-training),* a Wilcoxon Signed-Rank Test for two dependent samples (within-group comparisons) was performed due to the small number of participants in the follow-up assessment and the fact that most variables deviated from the normal distribution.

## Results

### Pre- vs post-training comparison of cognitive functions

**Figure 3:**
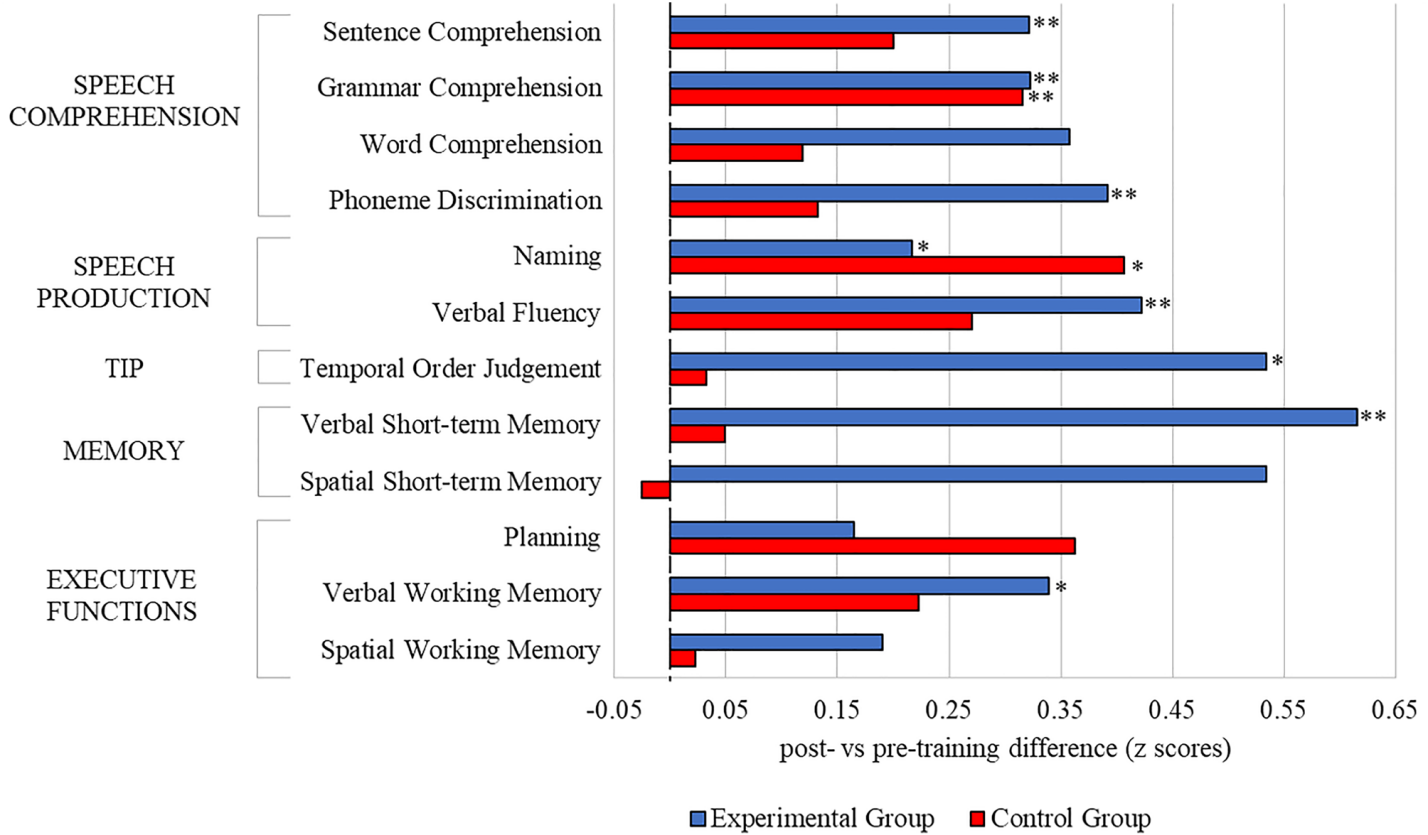
Difference in z scores (post-training minus pre-training) for participants in the two training groups. The z scores for both post-training and pre-training were referred to pre-training performance of all participants (across both groups). The following formula was implemented: difference in z scores = [(X2—M1)/SD1] −[(X1—M1)/SD1], where X1 and X2 refer to an individual score for particular test in pre-training and post-training assessment respectively, M1 and SD1 refer to mean and standard deviation for all participants in this test in pre-training assessment. Positive values (right side from the 0 point) correspond to improved performance. Negative values correspond to worsened performance (left side from the 0 point). The significant differences between *post-training* and *pre-training* scores are indicated by asterisks: *p < 0.05; **p < 0.01. Mean z score values (and standardised deviation) for ExpG and ConG for each test respectively: sentence comprehension: 0.32 (0.29) and 0.20 (0.58); grammar comprehension: 0.32 (0.61) and 0.32 (0.67); word comprehension: 0.36 (0.73) and 0.12 (0.61); phoneme discrimination: 0.39 (0.5) and 0.13 (0.67); naming: 0.22 (0.31) and 0.41 (0.70); verbal fluency: 0.42 (0.54) and 0.27 (0.71); TOJ: 0.53 (0.93) and 0.03 (0.5); verbal short-term memory: 0.62 (0.94) and 0.05 (0.81); spatial short-term memory: 0.53 (0.62) and − 0.03 (0.95); planning: 0.17 (0.97) and 0.36 (1); verbal working memory: 0.34 (0.58) and 0.22 (0.47); spatial working memory: 0.19 (0.58) and 0.02 (0.66).

The effect of experimental and control training was evaluated for particular tasks. The profile of changes in *pre-* vs *post-training* performance is given in Fig. 3.

#### Speech comprehension

- Sentence comprehension: the number of correct responses *post-training* (M = 37.85) was significantly higher (Z = − 3.068; p = 0.002) compared to *pre-training* (M = 33.69) in ExpG. The corresponding difference in ConG was nonsignificant (Z = − 1.072; p = 0.284; M = 29.17 vs M = 26.58 in *post-* and *pre-training*, respectively).
- Grammar comprehension: the number of correct responses *post-training* was significantly higher than *pre-training* (F(1;32) = 8.465; p = 0.007; η^2^ = 0.209). The interaction between the group and measurement was nonsignificant (F(1;32) = 0.001; p = 0.975; η^2^ < 0.001).
- Word comprehension: the difference in the number of correct responses was nonsignificant between *post-training* (M = 12.39) and *pre-training* (M = 11.44) in ExpG (Z = − 1.87; p = 0.062). This difference was also nonsignificant in ConG (Z = − 0.66; p = 0.509; M = 12.25 and M = 11.94 for *post-* and *pre-training*, respectively).
- Phoneme discrimination: the number of correct responses *post-training* (M = 22.44) was significantly higher (Z = − 2.885; p = 0.004) than *pre-training* (M = 20.78) in ExpG. The corresponding difference in ConG was nonsignificant (Z = − 0.537; p = 0.591, M = 19.94 vs M = 19.38 in *post-* and *pre-training*, respectively).

#### Speech production

- Naming: the number of correct responses *post-training* (M = 6.44) was significantly higher (Z = − 2.003; p = 0.045) than *pre-training* (M = 5.56) in ExpG as well as in ConG (Z = − 2.458; p = 0.014; M = 5.43 vs M = 3.79 for *post-* and *pre-training* respectively).
- Verbal fluency: the number of produced words was significantly higher (Z = − 2.626; p = 0.009) *post-training* (M = 13.56) than *pre-training* (M = 10.56) in ExpG. The corresponding difference in ConG was nonsignificant (Z = − 1.160; p = 0.246; M = 11.23 vs M = 9.31 in *post-* and *pre-training*, respectively).

#### Temporal information processing

- Temporal order judgement: TOT values in ExpG were significantly lower (Z = -2.154; p = 0.031) *post-training* (M = 126.68) than *pre-training* (M = 166.24). The corresponding difference in ConG was nonsignificant (Z = -0.517; p = 0.605; M = 120.17 vs M = 122.58 in *post-* and *pre-training*, respectively).

#### Memory

- Verbal short-term memory: the number of correctly reproduced sequences in ExpG was significantly higher (Z = − 2.599; p = 0.009) *post-training* (M = 4.69) than *pre-training* (M = 3). The corresponding difference in ConG was nonsignificant (Z = − 0.205; p = 0.837; M = 4.33 and M = 4.20 for *post-* and *pre-training,* respectively).
- Spatial short-term memory: the number of correctly reproduced sequences in *post-training* and *pre-training* did not differ significantly (F(1;31) = 3.39; p = 0.075; η^2^ = 0.098). The interaction between measurement and group was nonsignificant F(1;31) = 4.11; p = 0.051; η^2^ = 0.117).

#### Executive functions

- Planning ability: the difference between the number of correctly solved trials was nonsignificant (Z = − 0.595; p = 0.552) between *post-training* (M = 3.80) and *pre-training* (M = 3.47) in ExpG. The corresponding difference was also nonsignificant in ConG (Z = − 1.35; p = 0.177; M = 4.67 and M = 3.93 for *post-* and *pre-training*, respectively).
- Verbal working memory: the number of correctly reproduced sequences in ExpG was significantly higher (Z = -2.132; p = 0.033) *post-training* (M = 3.56) than *pre-training* (M = 2.75) The corresponding difference in ConG was nonsignificant (Z = -1.705; p = 0.088; M = 3.27 and M = 2.73 for *post-* and *pre-training*, respectively).
- Spatial working memory: the number of correctly reproduced sequences *post-training* and *pre-training* did not differ (F(1;31) = 0.961; p = 0.335; η^2^ = 0.030). The interaction between measurement and group was nonsignificant F(1;31) = 0.598; p = 0.445; η^2^ = 0.019).

### Stability of changes

The stability of changes was assessed in ExpG and ConG on the basis of comparisons between *follow-up* vs *post-training*. The results of these comparisons are given in Table 4. In ExpG, the differences between *follow-up* and *post-training* assessments for all measured cognitive functions were nonsignificant. This suggests that the effects of experimental training were stable over a 3-month period. In ConG, most cognitive measures remained unchanged; however, the number of correct responses on the Phoneme Discrimination Test and Spatial Short-term Memory Test were higher at *follow-up* compared to *post-training*.

**Table 4:** The stability of training effects over a 3-month period in ExpG and ConG (*follow-up* vs *post-training* assessment comparisons).

	ExpG (n = 10)	ConG (n = 9)
Speech comprehension		
Sentence comprehension	Z = − 0.141 p = 0.888	Z = − 1.289 p = 0.197
Grammar comprehension	Z = − 0.259 p = 0.796	Z = − 0.781 p = 0.435
Word comprehension	Z = − 0.857 p = 0.391	Z = − 0.544 p = 0.586
Phoneme discrimination	Z = − 0.962 p = 0.336	**Z = − 2.032** **p = 0.042**
Speech production		
Naming	Z = − 1.802 p = 0.072	Z = − 0.276 p = 0.783
Verbal fluency	Z = − 1.131 p = 0.258	Z = − 0.405 p = 0.686
TIP		
TOJ	Z = − 0.533 p = 0.594	Z = − 0.415 p = 0.678
Memory		
Verbal short-term memory	Z = − 0.844 p = 0.399	Z = − 1.066 p = 0.286
Spatial short-term memory	Z = − 0.214 p = 0.831	**Z = − 2.251** **p = 0.024**
Executive functions		
Planning	Z = 0.0 p = 1.0	Z = − 1.200 p = 0.230
Verbal working memory	Z = − 1.179 p = 0.238	Z = − 0.108 p = 0.914
Spatial working memory	Z = − 0.359 p = 0.719	Z = − 1.845 p = 0.065

The significant results are bolded.

### Summary of results

The application of the experimental training in PWA improved all assessed functions, apart from word comprehension, spatial short-term and working memory and planning ability. On the other hand, following the control training, significant improvement was observed only in grammar comprehension and naming. All reported improvements were relatively stable for 3 months after the experimental and control trainings, apart from the significant improvement in ConG in phoneme discrimination and spatial short-term memory.

## Discussion

The present study measured the effects of the Dr. Neuronowski^®^ training software (experimental training) and linguistic training (control training) on Temporal Information Processing (TIP), language skills, and other cognitive functions in people with aphasia (PWA). The results indicated that, after the experimental training, improvements could be observed in several non-linguistic functions (TIP, verbal short-term and working memory) as well as in several linguistic functions: sentence comprehension, grammar comprehension, fluency, naming, and phoneme discrimination (Fig. 3). In contrast, the control training resulted in significant improvement only in grammar comprehension and naming (Fig. 3). As the level of pre-training performance might be considered to be comparable between both groups (nonsignificant statistical between-group differences, despite some discrepancies between means of some variables in favour of ExpG (see Table 2), the differences in the effects of the trainings indicates divergent clinical effects.

Several cognitive functions were exercised in the experimental training, with a particular emphasis on TIP, which resulted in the lowering of participants’ temporal order thresholds (TOT), enhancement of phoneme discrimination (i.e., increased number of correct responses on the Phoneme Hearing Test), as well as improved maintenance of verbal stimuli in short-term memory (i.e., higher score on the Verbal Memory Test Forwards) in the post-training assessment. This improvement of the directly-trained functions may be considered as near transfer—improvement in tasks structurally similar to those trained^46^.

However, as phoneme hearing is also heavily rooted in millisecond TIP^22^, we cannot exclude the possibility that the enhancement of phoneme discrimination may be also a result of TIP–phoneme discrimination domain transfer. Moreover, we speculate that improvement of verbal short-term memory—that is to say, the increase of the number of verbal elements maintained in memory—further facilitated manipulation of those elements, resulting in amelioration of verbal working memory following experimental training.

Of particular interest is the improvement of the following linguistic functions in ExpG: sentence comprehension, grammar comprehension, fluency, and naming. Experimental training did not provide practice for those functions, which may suggest more complex mechanisms of transfer. PWA improved in the comprehension of longer language units, as evidenced in better performance on sentence comprehension and grammar comprehension in post-training vs pre-training assessment. Similar effects were reported by Szeląg et al.^21^ and Szymaszek et al.^48^ following the trainings based solely on TIP exercises. Additionally, Oron et al.^61^ documented a link between millisecond TIP and sentence comprehension. They emphasised that decoding the meaning of complex sentences requires high working memory load^40,62,63^. This is also in line with studies showing the amelioration of sentence comprehension following working memory training in PWA^41,42,44^. It has also been shown that working memory is associated with TIP^38,55^. Thus, the observed increased number of correct responses on the Token Test (sentence comprehension) and Grammar–Sentence Comprehension Test (grammar comprehension) in ExpG may be caused by improvement of verbal working memory capacity as well as TIP ability (far-transfer effect).

It is an important and novel finding of this study that the Dr. Neuronowski^®^ training method has significant clinical benefits for the amelioration of speech production functions—in particular, for naming and verbal fluency. Both naming and verbal fluency require the engagement of working memory and, furthermore, verbal fluency is additionally based on processing speed and auditory attention^64–66^. The experimental training might have enhanced these functions, which in turn resulted in improvement of speech production.

This improvement of untrained functions following the experimental training may indicate a far-transfer effect—that is, the transfer of training benefits to a different domain that shares underlying mental processes with the trained one^46,47^. On the basis of von Steinbüchel and Pöppel’s model^35^ (see Introduction), we suspect that the enhancement of TIP may result in the improvement of other, not directly trained functions, such as language. We postulate that this effect may be mediated by improvements in working memory. However, further studies are needed to elucidate this hypothesis.

After the Dr. Neuronowski^®^ training, no significant improvement was observed in word comprehension, spatial short-term and working memory, or planning ability. This may be due to different factors. We postulate that a ceiling effect was observed in the pre-training assessment of word comprehension, which resulted in little room for improvement by the post-training assessment. The factors which likely contributed to improvements in verbal short-term and working memory—TIP exercises, phonological hearing, and maintenance of verbal stimuli in memory—did not, by their nature, provide any transferable benefits for the visuo-spatial domain. Finally, referring to a previous study with a healthy group^67^, we expected that improved TIP would enhance the organisation and coordination of sequences of events, resulting in better performance on the planning task. However, we did not observe any significant improvement here. As planning is a very complex function, performance of the Tower of London task greatly engages one’s spatial working memory^68^, which is impaired in PWA and did not improve here, potentially contributing to the lack of significant improvement.

It is, however, worth noting that, despite the emphasis on TIP, particular modules of Dr. Neuronowski^®^ engaged other cognitive functions, such as short-term memory or executive functions. It is difficult to disentangle the influence of each trained function on the final improvement following experimental training. However, the inclusion of TIP in the majority of modules, as well as the findings of previous studies^21,48^ in which the training of TIP alone improved linguistic functions, suggest that the observed effects may be attributable to the improvement of TIP.

In contrast, the control training resulted in improvement of only grammar comprehension and naming, which were directly practised. No significant improvement was observed in several linguistic measures: sentence comprehension, verbal fluency, and phoneme discrimination. As previously noted, sentence comprehension (measured with the Token Test) requires non-linguistic functions (e.g., working memory, which was not trained in this group), and therefore simple practice in sentence comprehension may have been insufficient to improve performance in this test. Similarly, tasks that assess verbal fluency, involving spontaneous generation of words from specific semantic categories, engage verbal working memory and semantic control as well as auditory attention and processing speed, which were not trained in the control group^66^. Phoneme discrimination also did not improve. While the experimental training involved exercises dedicated to practicing phoneme discrimination, the control exercise was more focused on the word and sentence level. We also did not observe any improvement in non-linguistic functions, such as TIP, verbal short-term and verbal working memory, and planning, which were not trained in this group.

According to Abikoff and Ramsey^69^, in addition to the transferability of the effects, the value of a training should be assessed based on the stability of the improvement. The performance in the follow-up assessment indicated the stability of the effects of both trainings. Most functions remained on a relatively stable level over the 3-month period in both groups—with the exception of phoneme discrimination and spatial short-term memory which further improved in the control group. Although PWA did not participate in any therapy during this period, this may be due to further restoration of brain functions following the stroke or as a consequence of spontaneous daily activities^70^.

It is worth mentioning that we expected more widespread linguistic improvement in the control group. However, we speculate that the standardising and adjusting of experimental settings for both trainings resulted in a reduction of patient–therapist communication that occurred, which is typically associated with traditional speech training. This may have also hindered the effectiveness of this training^71^.

It is important to note that unequal number of patients participated in the following assessments (pre- and post- vs follow-up) was the limitation of the current study. Consequently, the main analyses were conducted only on the post- vs pre-training assessments as the follow up assessment was completed by a smaller number of patients (see “Methods”), which did not allow us to include all three measurements in the single statistical model. Another limitation is that, due to relatively small sample sizes and the large heterogeneity typically observed in studies involving clinical populations, correction for multiple comparisons was not applied. This decision was made to avoid potential non-detection of existing effects.

In summary, the current results are in line with previous studies regarding the use of training of TIP alone in PWA^16,21,48^. The use of the comprehensive Dr. Neuronowski^®^ cognitive training, which addressed a variety of cognitive functions with major emphasise on TIP, proved to be effective for restoring impaired linguistic and non-linguistic functions in PWA. Clinically, the results demonstrate that Dr. Neuronowski^®^ may be considered as a new tool for ameliorating linguistic and non-linguistic processing in PWA, and may therefore find use in the future clinical practice.

## Data Availability

The datasets generated and/or analysed during the current study are available from the corresponding author on request.

## Abbreviations

PWA: People with aphasia
ExpG: Experimental group
ConG: Control group
TIP: Temporal information processing
TOT: Temporal-order threshold
TOJ: Temporal-order judgement
